# Abiotic, present-day and historical effects on species, functional and phylogenetic diversity in dry grasslands of different age

**DOI:** 10.1371/journal.pone.0223826

**Published:** 2019-10-15

**Authors:** Rocío Belinchón, Lucie Hemrová, Zuzana Münzbergová

**Affiliations:** 1 Department of Botany, Faculty of Science, Charles University, Prague, Czech Republic; 2 Institute of Botany CAS, Průhonice, Czech Republic; Helmholtz Centre for Environmental Research - UFZ, GERMANY

## Abstract

Many grasslands have disappeared over the last century as a result of anthropogenic land use intensification, while new patches are emerging through abandonment of arable fields. Here, we compared species (SD), functional (FD) and phylogenetic (PD) (alpha) diversity among 272 dry grassland patches of two age-classes: old and new, with the new patches being dry grasslands established on previous intensively managed fields during the last 30 years. We first compared SD, FD and PD, between patches of different age. Then, we performed generalized linear models to determine the influence of abiotic, present-day and historical landscape configuration variables on SD, FD and PD. By measuring abiotic variables, we explained the effect of environmental filtering on species diversity, whereas the present-day and historical landscape configuration variables were included to describe how the spatial and temporal configuration of the patches influence patterns of species. Finally, we applied partial regressions to explore the relative importance of abiotic, present-day and historical variables in explaining the diversity metrics and how this varies between patches of different ages. We found higher SD in the old compared to the new patches, but no changes in FD and PD. SD was mostly affected by abiotic and present-day landscape configuration variables in the new and the old patches, respectively. In the new patches, historical variables explained variation in the FD, while present-day variables explained the PD. In the old patches, historical variables accounted for most of the variation in both FD and PD. Our evidence suggests that the relative importance of assembly processes has changed over time, showing that environmental filtering and changes in the landscape configuration prevented the establishment of species in the new patches. However, the loss of species (i.e. SD) is not necessarily linked to a loss of functions and evolutionary potential.

## Introduction

The impact of anthropogenic land use changes on local habitat conditions and landscape configuration often cause species loss and community composition change, affecting the regional persistence of plant species [1, 2]. After the World War II, land expropriation in Central and Eastern European countries together with the rise of intensive agriculture caused massive abandonment of traditional management [3, 4]. While many grassland habitats are being lost due to land use intensification or shrub encroachment, new patches are also emerging through abandonment of arable fields [5], representing new potential habitats for grassland species [6, 7]. Not all species are, however, able to spread into the new grassland patches due to strong alterations of abiotic conditions of these habitats compared to fields abandoned in previous time periods (e.g. [8–10]) or due to changes in patch configuration within the landscape [6, 11]. Therefore, the regional persistence of grassland species might depend not only on the rate of loss of their habitat, but also on the species’ ability to colonize the new patches such as abandoned fields [8].

Grassland species composition is influenced by environmental and dispersal limitations, because both are key components of the colonization process [11, 12]. Disentangling their relative importance is still a challenge in community ecology and aims to provide a more comprehensive understanding of the mechanisms that control over community assembly [11]. Two main drivers are generally considered to act as filters for grassland species: abiotic and landscape configuration variables [11]. Abiotic conditions, mostly in relation to resource requirements and physiological tolerance, are the key drivers determining the establishment success of grassland species [11, 13]. Specifically, microclimate and edaphic conditions strongly act as environmental filters affecting germination and seedling survival in grasslands [14] and affect competitive relationships among species [10]. Landscape configuration characterized by patch size and patch connectivity, at present and in the past, represents the variables affecting species potential to colonize the patches and establish viable populations in these habitats [15]. On one hand, the present-day landscape configuration has repeatedly been shown to influence colonization and extinction probabilities of species (e.g. [6, 16, 17]). For instance, large patches typically have increased habitat heterogeneity, which would support larger population sizes (e.g. [18, 19, 20]). Furthermore, isolated patches are more likely to be dispersal limited than connected patches and contain fewer species simply due to distance-dependent dispersal rates of species [15]. Moreover, landscape configuration may also have a historical component that describes the change in landscape configuration over time [19, 21]. This includes how the area and connectivity of the patches have changed in different past time periods. Previous studies have shown that diversity patterns reflect historical landscape configuration because grassland species experience long time-lags between anthropogenic land use change and community response (e.g. [22–25]). Also, patch age might influence species diversity, because the probability of a patch being colonised increases with the time for which the patch has been available for colonisation [6, 26].

Biodiversity is made up of three main components (i.e. species, functional and phylogenetic diversity–hereafter ‘SD’, ‘FD’ and ‘PD’ respectively) that complement ecosystem functioning and services [27]. SD is the number of different species that are represented in a community. FD reflects the degree of trait dissimilarity among species indicating species ability to use resources and disperse (e.g. including ecological, morphological, physiological or phenological traits) [28, 29]. PD measures the evolutionary relationships among species within a community [30]. Traditionally, most studies determining biodiversity patterns only focus on SD. However, SD alone cannot appropriately describe the mechanisms involved in species co-existence [30, 31], and, thus ignores the fact that not all species within a community are functionally or phylogenetically equivalent [32, 33]. For instance, species succession following anthropogenic land use change can lead to communities with equal SD that greatly differ in FD and PD [34, 35].To fill this gap, studies have been incorporating FD and PD to complete the information provided by SD by adding insights into community functioning and community evolutionary history [30,36]. The results show that the combination of different diversity indices not only better addresses trends in biodiversity [37], but it also provides a deeper understanding of the influence of species dispersal, competition ability and evolutionary distinctiveness in determining patterns of community assembly [28, 29, 38].

Dry grasslands provide an excellent model system to determine diversity patterns because their communities host small-scale rich assemblages of vascular plants [8, 39]. They are considered one of the most biodiverse habitats with a high conservation value (i.e. priority habitats) in Europe [40], including a high number of threatened species [10]. Previous studies of grassland succession on formerly arable fields have mostly evaluated changes in species richness (e.g. [12]), as well as the influence of environmental and dispersal filtering in species composition [4, 41]. However, during the last years, there is a growing consensus that changes in grasslands during succession are linked to ecological and evolutionary differences among species, and recent studies are incorporating functional or phylogenetic traits into the studies [42, 43]. Yet, the studies that include FD are mostly based on descriptive individual traits at species level and are not incorporating trait dissimilarity into comparison of communities (e.g. [6, 11, 44, 45]), while grassland PD studies are scarce [43, 46–48]. Indeed, studies that simultaneously evaluate multiple spatio-temporal variables on different diversity facets are unusual, with the subsequent difficulty to elucidate the mechanisms governing the grassland communities. For example, Purschke *et al*. [47] has quantified taxonomic, functional and phylogenetic diversity changes during succession in 270-year-long arable-to-grassland chrono sequence in Sweden. They found that late successional communities contained more functionally and phylogenetically divergent species than early successional stages. Although their study was based on multiple diversity facets (i.e. SD, FD and PD), abiotic and/or landscape configuration variables were not evaluated as drivers of community assembly. In a second study, Purschke *et al*. [21] evaluated whether the relative importance of dispersal and persistence traits on functional diversity is explained by the present-day and historical landscape configuration in the successional grassland landscape, but there is no information regarding the importance of environmental filtering. Or for example, Saar *et al*. [49] examined the species assembly in relation to species traits comparing grasslands with differing habitat history but there is no information about changes in SD or drivers generating different assembly patterns. To gain a deeper understanding of the processes governing community assembly during succession, we need to i) estimate whether ecological or evolutionary processes are driving community assembly and ii) assess the relative importance of environmental filtering and landscape configuration in shaping the community structure.

Here, we examined patterns in SD, FD and PD (alpha diversity) by comparing dry grassland communities of two different ages (old vs new patches; “new patches” are grasslands established on previous intensively managed fields during the last 30 years and “old patches” represent grasslands with 44–221 years of continuity) in a fragmented landscape. Specifically, we determined the influence of environmental filtering by exploring the effect of abiotic variables that prevent the establishment of species in a location. In addition, we studied the extent to which the spatial and temporal landscape patterns are affecting plant diversity by determining the present-day and historical landscape configuration of the dry grassland patches. A previous study conducted in the same area has already revealed that dispersal traits and habitat preferences are determining the distribution of single dry grassland species [6]. They found that rapidly growing species with long-term persistent seed bank and high nutrient requirements occur in young, large and connected patches, whereas species specialists (i.e. stress-tolerant species with narrower habitat requirements based on niche width estimation [9]) occurred in older, historically large and currently more isolated smaller patches [6]. However, we lack information on i) whether the changes in the distribution of single species translate into changes in SD, FD and PD, ii) what are the variables driving this differentiation and iii) whether patch age influences our results.

In particular, we asked the following questions: (1) What is the effect of abiotic, present-day and historical landscape configuration variables on SD, FD and PD?, (2) How do the relative contributions of abiotic, present-day and historical variables differ between the three diversity metrics?, (3) How do our findings vary between old and new patches? We expect that abiotic conditions (i.e. environmental filtering) will be dominant in the new patches as their communities are not at equilibrium. In addition, we predict that present-day landscape configuration variables will exhibit stronger effects in the new communities–but not in the old ones, as old patches are more determined by the past. As old patches have been available for colonization for longer time and abiotic conditions are more heterogeneous, we hypothesize a higher SD compared to the new patches. Besides, the species within the old patches will be less related to each other than species from the new patches, which will be translated into higher FD and PD in the old patches.

## Materials and methods

### Ethical statement

No specific permissions were required for performing this study. This study was purely observational so did not involve collection of any plant material. All the fieldwork was done on a freely accessible land, so no permission to enter specific locations was required.

### Study area and patches

The study area (8 × 8 km) is situated in northern Bohemia, Czech Republic (NW corner: 50°33’19” N, 14°15’6” E, SE corner: 50°29’45” N, 14°22’31” E; see [7] for details) (S1 Fig). The climate is continental with a mean annual temperature of 7.7°C, and annual rainfall of 612 mm. The landscape is a mosaic of calcareous dry grasslands (alliance *Bromion erecti*, [50]), surrounded by shrubs and large agricultural fields. The area is associated with a long tradition of agriculture. Many new grasslands raised in the area after abandonment of the fields that were intensively managed since the mid-20^th^ century (S1 Table and S1 Fig). At present, agricultural land covers more than 70% and dry grasslands occur in small fragments totaling 4% of the study area [6].

For this study, we used data on plant species composition collected at 272 dry grassland patches in 2009 for a purpose of a previous study (see [6] for data collection and S2 Table for the list of the species). A dry grassland patch was defined as a site with visually homogeneous vegetation with at least three dry grassland species and separated from other patches by a visual topographic barrier such as a small ditch or change of slope from very steep to flat [6]. All our dry grasslands are a result of natural succession, including the combination of seed bank and seed dispersal, and therefore none of the sites have been artificially seeded. The presence of dry grassland species was recorded in the grassland patches by detail search for all grown-up species, but not determining the seedlings. From the previous study, we excluded thirteen dry grassland patches that were not big enough to get relevant data from historical maps (< 100 m^2^). All 272 selected dry grassland patches were classified according to their continuous existence in the landscape with the help of GIS using historical maps from 1843, 1954 and 1980 [6]. Patches present on the 1980 map were classified as ‘old’ dry grasslands (n = 216) and patches absent at the 1980 map represented ‘new’ patches (n = 56) established on previously managed fields (S1 Fig). Both ‘old’ and ‘new’ patches are comparable in management regime as they are currently abandoned and have not been subjected to any management for the last 40 years.

### Abiotic, present-day and historical landscape configuration variables

To study the influence of environmental filtering on dry grassland communities, we measured **abiotic conditions** (Table 1). For each dry grassland patch, we measured ten abiotic variables that control water and light availability in the patches. We used ArcGIS 10.2 to calculate mean values of ‘slope’, ‘potential direct solar irradiation’ from December to June (PDSI), ‘topographic wetness index’ (TWI) and ‘relative elevation’ from the main water course (Elevation) based on a digital elevation model (DEM) with a 10 m grid size. To avoid problems with collinearity, we carried out Pearson correlation tests among all explanatory variables. We identified strong correlations (Pearson R > 0.5) among solar irradiation variables and decided to include only solar irradiation in June in further analyses (S2 Fig), as summer drought highly influences species richness and composition of the dry grassland communities [7].

**Table 1:** List of variables assembled for old and new dry grassland patches in this study.

Variable	Units	Type
Abiotic		
TWI* [51]		Topographic wetness index to describe spatial soil moisture patterns
Slope	degrees	Steepness
Elevation	m	Elevation above the main water course
PDSI_(December to June)		Potential direct solar irradiation of every month from December to June
Present-day landscape configuration (2009)		
Log (A)	m^2^	Logarithm of area of present-day grasslands
I_2000_		Isolation based on the present-day area of the surrounding dry grasslands
Historical landscape configuration		
I_1843_		Isolation based on the area of the surrounding potential grassland habitats in 1843
I_1954_		Isolation based on the area of the surrounding potential grassland habitats in 1954
I_1980_		Isolation based on the area of the surrounding potential grassland habitats in 1980
A_1843_		Area of the patch calculated as percentage of its present-day area covered by PGH in 1843
A_1954_		Area of the patch calculated as percentage of its present-day area covered by PGH in 1954
A_1980_		Area of the patch calculated as percentage of its present-day area covered by PGH in 1980
Age	years	Number of years of continuous existence of a patch in the landscape

*non-dimensional index calculated as *TWI* = *ln* (*As/tan b*), where As is the specific catchment area (the cumulative upslope area draining through a cell divided by the contour width), and b is the local slope. PGH, potential grassland habitats.

To characterize **present-day landscape configuration**, we calculated for each dry grassland patch its area and isolation (Table 1) using digital maps in ArcGIS 10.2 (see [6]). Both variables have been shown to influence colonization and extinction probabilities of species (e.g. [6, 16, 17]). ‘Present-day patch area’ was determined as the size of the dry grassland patch. ‘Present-day isolation’ was calculated using Eq 1 (I_j_: calculated using the area of target patch) provided by [7]. Values were higher for more spatially isolated sites, when the source dry grasslands were smaller.

$$I_{j}=-log\;\sum_{k=1}^{n}\left[\left(\frac{A_{k}}{d_{jk}^{2}}\right)\right],j\neq k$$ {Eq 1}

where *I*_*j*_ is the isolation of a patch *j*, *k* represents all of the surrounding patches within a 0.5 km distance of patch *j*; *A*_*k*_ is the area of the patch *k* (Eq 1); We measured *d*_*jk*_ as edge-to-edge distance. We considered the circuit with a radius of 0.5 km as having the best fit for calculating isolation in dry grasslands [52].

As **historical** changes in the **landscape configuration** also affect present-day communities, we calculated the patch area and isolation from past time-periods (Table 1). We used digital maps from 1843, 1954 and 1980 past time-periods, which were created in a previous study [6], which contain information on abandoned fields, pastures and meadows, considering them as potential grassland habitats (PGH, S1 Table and S3 Fig). As the real boundaries of present dry grassland patches hosting homogeneous vegetation could not be identified in historical maps, we selected PGH based on cadastral information [6]. We then calculated abiotic variables (see above) defining the old dry grassland patches, including minimum and maximum values for each abiotic variable (S3 Table). From the total PGH, we only selected those PGH patches that fit abiotically to old dry grasslands`abiotic variables, while removing all other PGH when abiotic variables were out of range limit ± SE (S3 Table). In this way, we excluded PGH that were unlikely to host dry grassland species, e.g. floodplains or steep north-facing slopes with no sunlight, among others.

Next, adapting a method from [6], the ‘historical patch area’ was calculated as the percentages of its present-day area covered by PGH in individual past time periods (i.e. in the 1980s, 1954 and 1843). Then, we calculated the ‘historical patch isolation’ of each present-day patch to PGH in each time-period separately using Eq 1 (see above). Values of historical isolation are higher for smaller grassland patches which are further apart from PGH. As we found strong correlation among historical variables, I_1954_ and A_1954_ were excluded from subsequent analyses (Pearson R > 0.6; S4 Fig). We also used ‘patch age’ variable from our previous study [6] that was determined as the time the grassland patch has been continuously present in the landscape (i.e. grassland continuity). A patch newly appearing on a given map was assumed to have occurred in the middle of the period between the creation of this map and the preceding map. The age of the patches ranged from 13–221 years, with the old patches assigned to 44–221 years of dry grassland continuity.

### Species, functional and phylogenetic diversity

Measures of SD, FD and PD (alpha diversity) were calculated for each patch. To calculate **species diversity (SD),** we used the presence/absence data from 99 dry grasslands species collected in our previous study performed in the region [6]. The species list was a priori established based on the knowledge of the landscape [8, 39, 53], including species restricted to dry grasslands that are not occurring in other habitat types in the region and excluding the very common and very rare species as it would not be possible to obtain reliable estimates of their response to habitat conditions for the subsequent analyses (see S2 Table for a list of the species and their frequency). This is important as otherwise we would not be able to study the patterns in community assembly in dry grasslands. We then calculated species diversity (SD) as the total number of species per patch.

To calculate **functional diversity (FD),** we used mean pairwise dissimilarity (using the ‘melodic’ function supplied in [54]). Mean pairwise dissimilarity is the mean of the dissimilarities in functional distance between all pairs of species occurring within a site. This index was selected as it has been found to be insensitive to species diversity [54]. First, we compiled information on 12 functional traits related to the whole life-cycle of the species: phenology, seed and vegetative traits (Table 2 and S5 Fig). Phenology traits show the adaptive potential of species to the environmental conditions (i.e. beginning, duration and end of flowering). Seed traits are related to species’ dispersal ability (i.e. terminal velocity, rate of epizoochory, rate of endozoochory, seed mass and seed bank longevity). Vegetative traits reflect competitive ability and are linked to the persistence of species (i.e. clonality, perenniality, plant height and SLA). Most of the traits had been measured in previous studies [6, 53] and Průchová unpublished data. The missing data were added from the literature or databases (for details of trait estimation see Table 2) Second, to calculate trait dissimilarity distances, three different Gower distance matrices of species (i.e. for each group of traits: phenology, seed and vegetative traits) were used (using the function *gowdis* with the ‘podani’ extension to ordinal variables, package FD [55]). Third, we computed a final Gower-distance matrix combining the three previous matrices. Following the advice in [54], the matrix diagonal was disregarded in functional distance matrices. All the calculations were done using R 3.4.0.

**Table 2:** List of variables of functional traits and ecological preference for 99 dry grassland species considered in the study. *Not included in the calculation of functional diversity index. Vegetative traits may reflect competition ability. Phenology traits show the adaptive potential of species to the environmental condition. Seed traits are related to species’ dispersal ability.

Trait	Abbreviation	Scale, units or categories	Range	Data source
Functional traits* ***[60]***				
Vegetative traits				
Clonality	CLON	Binary: presence (1)/absence (0)	0/1	[56, 57]
Perennial	PER	Binary: presence (1)/absence (0)	0/1	[56, 57]
Plant height	PHEI	Continuous [Log (m)]	-1.12–0.13	[53], Průchová unpublished data
Specific leaf area	SLA	Continuous [mm^2^ mg^-1^]	7.56–39.35	[56], own measurements
Phenology traits				
Beginning of flowering	BFLOW	Ordinal (month)	3–8	[58]
Duration of flowering	MFLOW	Quantitative (month)	1–6	[58]
End of flowering	EFLOW	Ordinal (month)	4–10	[58]
Seed traits				
Rate of endozoochory	ENDO	Continuos: proportion between 0 and 1	0–1	Průchová unpublished data
Rate of epizoochory	EPI	Continuous; proportion between 0 and 1	0–1	[53], Průchová unpublished data
Seed bank longevity	SBL	Continuous; dimensionless index between 0 and 1	0–1	[56]
Seed mass	SMASS	Continuous [Log (mg)]	-3.30–1.43	[53], Průchová unpublished data
Terminal velocity	TV	Continuous [m s ^-1^]	0.26–4.30	[53], Průchová unpublished data
Ecological preference*				
Ellenberg’s values				
Light	LIGHT	Ordinal: categories 1–9	5–9	[50, 59]
Moisture	MOIST	Ordinal: categories 1–12	2–7	[50, 59]
Nutrients	NUT	Ordinal: categories 1–9	1–6	[50, 59]

* We only included traits with complete information for the 99 species as FD indices decline in reliability with missing trait data. Data on specific leaf area (SLA), plant height and seed mass were log transformed to decrease the effect of extreme values. Only the rate of epizoochory and endozoochory included missing data (NAs in 13 species, < 13% data). We could not obtain trait measures for the SLA and seed bank longevity for 6 and 10 species, respectively and, we used the mean trait value of the genus (see S2 Table).

To explore which traits or ecological indicators govern the filtering of species, we calculated community mean (CM), as the mean of the trait/ecological values in the community for each patch (function *functcomp* (with presence-absence matrix data), package FD [55]) in R 3.4.0. To do this, we used the 12 functional traits presented above and the 3 ecological preference indicators (Table 2). Ecological preference of the species was based on Ellenberg’s indicators (moisture, light and nutrients) (Table 2), and they were used to assess strategies for survival under specific environmental conditions. As we detected strong correlation between moisture and nutrient indicators, only moisture was considered in our CM analyses (Pearson R > 0.5; S5 Fig).

To calculate **phylogenetic diversity (PD)**, we extracted data on phylogenetic relationships between all the species from the DAPHNE database [61]. Data on *Globularia punctata* were not available and were replaced by its sister species *G*. *nudicaulis* [62] in the analysis (S6 Fig). We also used mean pairwise dissimilarity (see above) to calculate the mean of the dissimilarities in phylogenetic distance between all pairs of species occurring within a site. Phylogenetic distance matrix was calculated as the pairwise cophenetic distance of all species in the phylogeny (ape package) [63]; also, the matrix diagonal was disregarded [54]. All the calculations were done using R 3.4.0.

### Statistical analysis

All statistical models were performed using R 3.4.0. Our data consisted of patch types (old and new), three dependent variables (SD, FD and PD) and three sets of predictors (abiotic, present-day and historical landscape configuration) (S4 Table). We run all the statistical analyses separately for the old and the new patches. In this way, we explored if the determinants of SD, FD and PD operating in the old dry grasslands also hold in the new grassland patches.

To compare differences in means between the old and the new patches, we performed separate (one sample) t-tests for the explanatory variables (i.e. abiotic, present-day and historical landscape configuration variables) and our diversity metrics (i.e. SD, FD and PD). Also, we explored differences in means for functional traits and ecological preferences (i.e. calculated as community mean values–CM, described above) between the old and the new patches by a Welch two sample t-test. As multiple testing is involved and to avoid type I errors, we applied the false discovery rate analyses (FDR, see [64] for details).

To explore the relationships of SD, FD and PD to abiotic, present-day and historical landscape configuration variables, we performed generalized linear models (GLMs). Prior to analysis, in order to eliminate redundancy in our data, we performed a principal component analysis (PCA) separately in each predictor set (abiotic, present-day and historical variables). Variables in each predictor set were transformed to reduce outliers when necessary and scaled to unit variance to give them equal weights in the PCA. Then, we extracted the two first axes of each PCA to use as explanatory variables (Table 3). This also allowed for better comparison between the three sets of predictors as each was represented by the same number of variables (i.e. two axes). The first two PCA axes selected for each predictor set accounted for 72.35, 100 and 64.34% of the variation in the abiotic, present-day and historical dataset, respectively (Table 3). We then performed generalized linear models (GLMs) exploring variation in SD, FD and PD in relation to the two PCA axes selected for each predictor set. Variance partitioning was used to calculate the relative importance of each predictor set either alone or in combination based on GLM models. Adjusted fractions of total variation explained (TVE, in %) were estimated following the procedure of Peres-Neto et al. [65]. Finally, to determine which individual variables within each predictor set (abiotic, present-day and historical) were explaining the variation in SD, FD and PD, we performed independent GLMs (i.e. separately models for each predictor set). GLMs were fitted assuming a Poisson error distribution for species diversity and Gaussian distribution for functional and phylogenetic diversity [66]. To identify the independent model that “best” explained the variation in SD, FD and PD, we used Akaike’s information criterion (AIC). Variance partitioning was implemented in the modEvA package [67].

**Table 3:** Results of principal component analysis using abiotic, present-day and historical sets of variables.

	OLD PATCHES	
**Abiotic variables**	**PC1**	**PC2**
TWI	0.703	0.081
slope	-0.702	-0.082
elevation	0.086	-0.701
Pdsi_June	0.077	-0.703
Proportion of variance (%)	41.76	30.59
Cumulative Proportion (%)	41.76	72.35
**Present-day variables**	**PC1**	**PC2**
Log A	-0.707	-0.707
I_2000_	0.707	-0.707
Proportion of variance (%)	66.88	33.13
Cumulative Proportion (%)	66.88	100
**Historical variables**	**PC1**	**PC2**
Age	-0.451	0.081
I_1843_	0.504	-0.308
I_1980_	0.479	0.415
A_1843_	-0.357	0.663
A_1980_	-0.429	-0.534
Proportion of variance (%)	40.65	23.69
Cumulative Proportion (%)	40.65	64.34

TWI (topographic wetness index), PDSI_June (potential direct solar irradiation June), LogA (logarithm patch area), I_A_ (Isolation based on the area of the surrounding dry grasslands in 2000), Age (number of years of continuous existence), I_1843_ (Isolation based on the area of the surrounding potential grassland habitats in 1843), I_1980_ (Isolation based on the area of the surrounding potential grassland habitats in 1980), A_1843_ (Area of the patch calculated as percentage of its present-day area covered by potential grassland habitats in 1843), A_1980_ (Area of the patch calculated as percentage of its present-day area covered by potential grassland habitats in 1980).

## Results

### Species diversity

SD was 27% lower in the new compared to the old patches (paired t-test p < 0.001, Table 4). The averaged SD per patch was 32.37 (7–69) and 23.64 (3–45) in the old and the new patches, respectively (Table 4). *Agrimonia eupatoria*, *Brachypodium pinnatum*, *Fragaria viridis* and *Knautia arvensis* were the most common species across the localities, while *Campanula rotundifolia*, *Centaurea stoebe*, *Euphrasia rostkoviana* and *Filipendula vulgaris* were the rarest (S2 Table). We also found that 22 species were present in the old patches and absent in the new ones: *Anthericum ramosum*, *Artemisia campestris*, *Asperula tinctioria*, *Aster linosyris*, *Campanula glomerata*, *Cirsium panonicum*, *Coronilla vaginalis*, *Globularia punctata*, *Gymnadenia conopsea*, *Laserpitium latifolium*, *Linum flavum*, *L*. *tenuifolium*, *Listera ovata*, *Melampyrum cristatum*, *Onobrichis viciifolia*, *Peucedanum oreoselinum*, *Pulsatilla pratensis*, *Scabiosa canescens*, *Scorzonera hispanica*, *Seseli hippomarathrum*, *Sesleria albicans* and *Thesium linophyllon*. Many of these are, however, rare even in the old patches.

**Table 4:** List of abiotic, present-day and historical landscape configuration variables and diversity metrics for old and new patches. Data shows the results of a paired t-test between the old and the new patches with correction for multiple testing.

	Old patches	New patches		
	mean ± SD (max-min)	mean ± SD (max-min)	P-value threshold	FDR-adjusted
Number of patches	216	56		
Abiotic				
**TWI**	**8.17 ± 0.94 (5.97–11.63)**	**8.84 ± 1.36 (6.50–13.6)**	**0.023**	**0.002**
**Slope**	**12.54 ± 5.01 (0.96–28.4)**	**9.35 ± 4.16 (3.53–19.47)**	**0.011**	**< 0.0001**
Elevation	70.48 ± 34.26 (14.82–173.61)	69.43 ± 32.27 (23.00–160.23)	0.146	0.853
PDSI_June	5546.66 ± 208.12 (4635.90–5809.36)	5521.93 ± 190.83 (5082.78–5798.04)	0.038	0.518
Present-day landscape configuration				
Log (A)	3.32 ± 0.63 (2.02–5.27)	3.28 ± 0.82 (1.82–4.71)	0.042	0.809
I_2000_	-2.23 ± 1.77 (-5.4–1.42)	-1.98 ± 1.90 (-4.71–1.87)	0.035	0.518
Historical landscape configuration				
**I**_**1843**_	**-3.25 ± 1.13 (-6.75–0.37)**	**-2.63 ± 1.51 (-4.94–0.10)**	**0.027**	**0.010**
**I**_**1980**_	**-3.72 ± 1.17–7.91-(-0.01))**	**-2.89 ± 1.58 (-5.48-(-0.10)**	**0.019**	**0.001**
**A**_**1843**_	**0.24 ± 0.31 (0–1)**	**0.09 ± 0.24 (0–1)**	**0.015**	**< 0.0001**
**A**_**1980**_	**0.62 ± 0.38 (0–1)**	**0.03 ± 0.08 (0–0.52)**	**0.004**	**< 0.0001**
Diversity metrics				
**Species diversity**	**32.37 ± 11.81 (7–69)**	**23.64 ± 8.97 (3–45)**	**0.008**	**< 0.0001**
Functional diversity	0.24 ± 0.001 (0.19–0.28)	0.24 ± 0.01 (0.22–0.28)	0.05	0.853
Phylogenetic diversity	0.75 ± 0.02 (0.66–0.82)	0.74 ± 0.03 (0.63–0.80)	0.031	0.086

Bold variables were significantly different between old and new patches, according to a t-test to compare difference in means. We applied false discovery rate correction for multiple testing (FDR, *p* ≤ 0.05).P-value threshold, threshold values for declaring significance after multiple correction test; FDR-adjusted, p-value after adjust the original P-values so that they reflect the multiplicity correction; TWI, topographic wetness index; PDSI_June, potential direct solar irradiation in June; Log (A), logarithm of patch area; I_2000,_ Isolation based on the present-day area of the surrounding dry grasslands (higher values for more isolated sites, when the source dry grasslands were smaller); I_1843_, Isolation based on the area of the surrounding potential grassland habitats in 1843; I_1980_, Isolation based on the area of the surrounding potential grassland habitats in 1980; A_1843_, Area of the surrounding potential grassland habitats in 1843; A_1980_, Area of the surrounding potential grassland habitats in 1980

**Fig 1:**
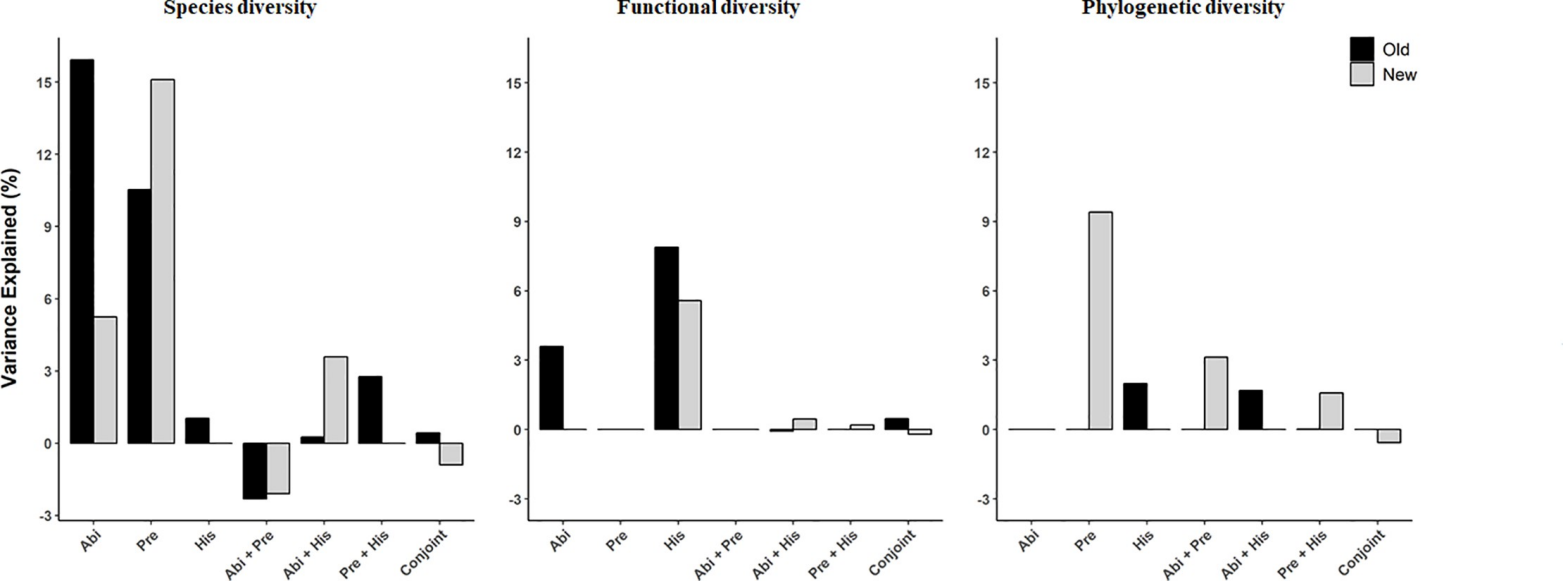
Variation partitioning of the three-diversity metrics (SD, FD and PD) explained by abiotic, present-day and historical variables. Variance partitioning was used to calculate the relative importance of each predictor set (using PCA axis as explanatory variables for abiotic, present-day and historical data) either alone or in combination based on GLM models. The black bars indicate the results for the old patches and the light grey bars indicate the results for the new patches. Abi, abiotic variables; Pre, present-day variables; His, historical variables; Conjoint, the effect of all predictors set together (i.e. abiotic + present-day + historical variables).

Variance partitioning showed that SD in the old patches was mostly affected by abiotic variables (15.8%), with present-day and historical landscape configuration variables explaining an additional 10.4% and 0.9% of the variance (Fig 1 and S7 Fig). Old patches occupied a broader range of environmental conditions compared to the new ones and were characterized by lower topographic wetness index and historical isolation, and higher slope and historical area (paired t-test p < 0.01, Table 4). For the new patches, the variation partitioning indicated that SD was mostly affected by present-day landscape configuration variables (15.1%), abiotic variables also accounted for an important 5.2% of the variation while historical variables exerted a negligible effect (Fig 1 and S7 Fig). The total variation by all three groups of explanatory variables (including abiotic, present-day and historical variables together) explained a similar percentage of variance in old and new patches (28.5% vs 22.8%) (Fig 1 and S7 Fig).

Results from GLMs indicated that SD in the old patches was affected positively by the slope and irradiation in June and negatively by TWI (Table 5). For the new patches, SD was only positively affected by the slope (Table 5). In relation to present-day landscape configuration variables, both area and isolation were important explaining SD in the new patches, whereas SD in the old patches was only positively affected by patch area (Table 5). In the case of historical landscape configuration variables, we observed in the old patches a higher SD in older and less isolated patches in 1843 and 1980, whereas SD in the new patches was higher in isolated patches in 1843.

**Table 5:** Results for generalized linear models (GLM). Data shows which variables within each predictor set (abiotic, present-day and historical landscape configuration) were explaining the variation in species (SD), functional (FD) and phylogenetic (PD) diversity in both the new and the old dry grassland patches.

	SD				FD				PD			
	old		new		old		new		old		new	
	z	p	z	p	z	p	z	p	z	p	z	p
**Abiotic**												
TWI	-3.557	***	-	-	-	-	-	-	2.043	*	-	-
Slope	6.081	***	4.679	***	3.517	***	-	-	-	-	-	-
Elevation	-		-	-	-	-	-	-	-	-	-	-
PDSI_June	3.644	***	-	-	-	-	-	-			-	-
**Present-day landscape configuration**												
LogA	10.56	***	2.353	*	-	-	-	-	-	-	-2.361	*
I_2000_	-	-	-4.369	***	-	-	-	-			-	-
**Historical landscape configuration**												
Age	5.150	***			-	-			-2.289	*		
I_1843_	-1.993	*	2.178	*	-2.575	*	-	-	-	-	-	-
I_1980_	-4.460	***	-	-	-	-	-2.138	*	-	-	-	-
A_1843_	-	-			2.036	*			-	-	-	-
A_1980_	-	-	-	-	-	-	-	-	-	-	-	-

TWI, topographic wetness index; PDSI_June, potential direct solar irradiation in June; LogA, logarithm of patch area; I_2000,_ Isolation based on the present-day area of the surrounding dry grasslands (higher values for more isolated sites, when the source dry grasslands were smaller); Age, number of years of continuous existence (“Age” variable was not included in the models for the new patches—they all have the same number of years of continuous existence); I_1843_, Isolation based on the area of the surrounding potential grassland habitats in 1843; I_1980_, Isolation based on the area of the surrounding potential grassland habitats in 1980; A_1843_, Area of the surrounding potential grassland habitats in 1843; A_1980_, Area of the surrounding potential grassland habitats in 1980.

*p-*values: *** 0.001, ** 0.01, * 0.05.

### Functional diversity

We found no significant difference in FD between the old and the new patches (paired t-test p = 0.85, Table 4). We found that FD was positively correlated to SD in the old patches (Pearson correlation test, r = 0.37, p < 0.01), although there was no significant relationship in the new patches (Pearson correlation test, p = 0.96).

**Fig 2:**
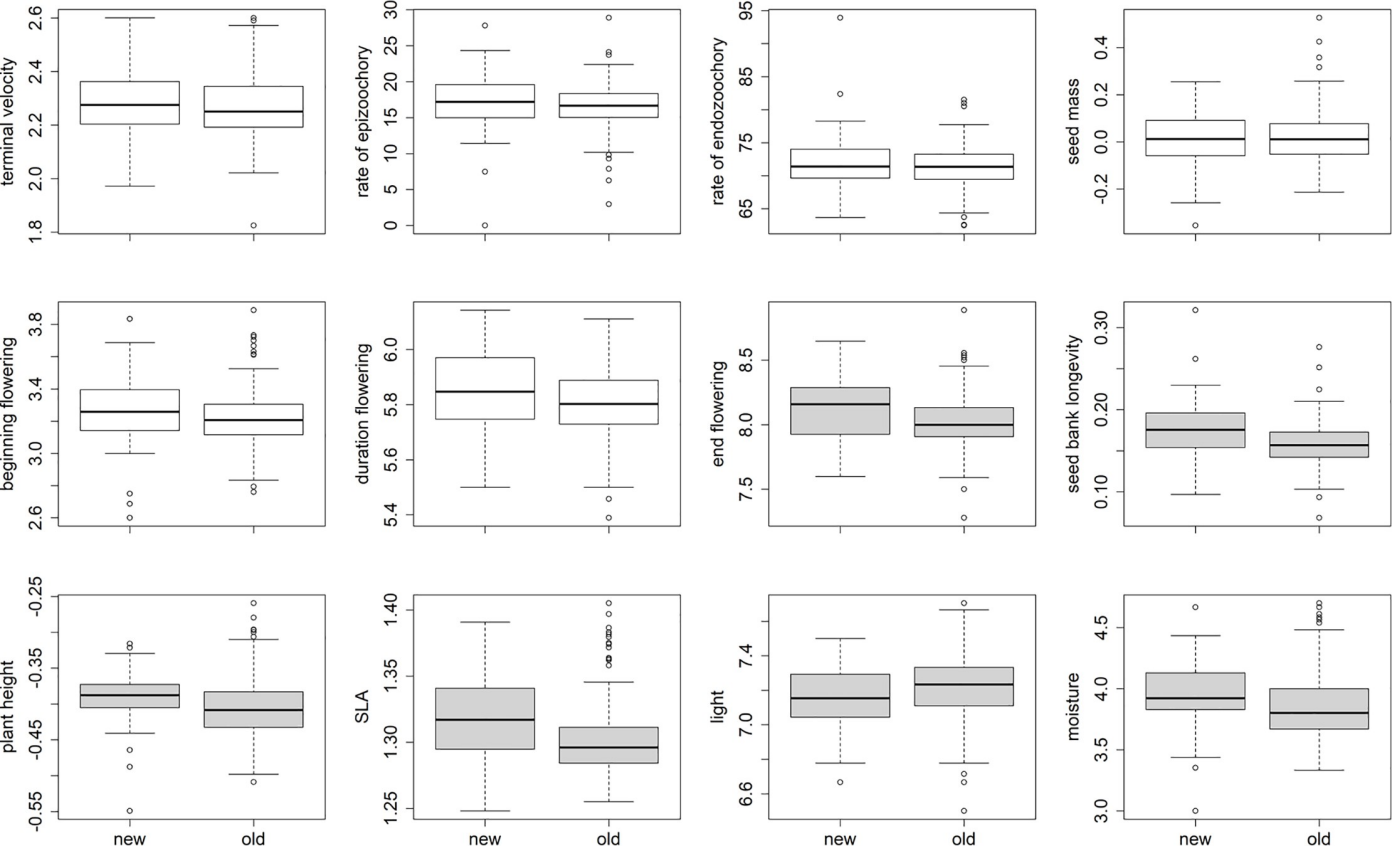
Community mean (CM) for traits and ecological preference variables used in this study. The difference between old and new patches was tested by a Welch two sample t-test (*p*-value < 0.05). Grey bars are significantly different between the new and the old patches.

Comparisons of community mean traits (CM) showed that SLA, plant height, end of flowering and seed bank longevity were higher in the new patches (Fig 2). However, there was no significant difference in CM for rate of epizoochory, rate of endozoochory, seed mass, terminal velocity, months of flowering and beginning of flowering. We also found that ecological preference in the new patches was characterized by lower light and higher moisture levels compared to the old patches (Fig 2).

Historical and abiotic variables explained 7.9% and 3.6% of the variation in the old patches while present-day conditions did not have any significant effect in the old patches. Only historical variables had significant effect in the new patches and explained 6.5% of the variation (Fig 1 and S7 Fig). The final combined model (including abiotic, present-day and historical variables together) explained 11.3% and 7% of variance in the old and the new patches, respectively (Fig 1 and S7 Fig). Results from GLMs showed higher FD in the steep old patches with large area in 1843 that were also connected in 1843, whereas FD was only negatively affected by isolation in 1980 in the new patches (Table 5).

### Phylogenetic diversity

PD was not significantly different in the old compared to the new patches (paired t-test p = 0.086, Table 4). PD was significantly correlated to SD in both new and old patches (Pearson correlation test, r = 0.35 and r = 0.33, p < 0.001). The average number of genera per patch type was 29.06 (7–54) and 21.78 (3–37) for the old and the new patches, respectively. We found that 12 genera that were present in the old patches and absent in the new ones: *Anthericum* spp., *Gymnadenia* spp., *Listera* spp., *Sesleria* spp., *Pulsatilla* spp., *Onobrychis* spp., *Thesium* spp., *Globularia* spp., *Scorzonera* spp., *Artemisia* spp., *Laserpitium* spp., *Seseli* spp.

Historical landscape configuration variables accounted for 2.0% of the variation in the old patches, while abiotic and present-day variables exerted a negligible effect on PD (Fig 1 and S7 Fig). In the new patches, present-day variables alone accounted for 9.4% of the variance, while abiotic and historical variables having negligible effects (Fig 1 and S7 Fig). Variance partitioning showed that the final combined model (including abiotic, present-day and historical variables together) accounted for 3.7% and 13.5% of variance in the old and the new patches, respectively (Fig 1 and S7 Fig). Results from GLMs showed a positive effect of the slope and negative influence of the isolation in 1843 were the most important variables in old patches, whereas we only found a negative effect of patch area in the new patches (Table 5).

## Discussion

Although SD was lower in the new patches, we found no evidence for a parallel decrease in FD and PD. Instead, the new patches were more functional and phylogenetic diverse compared to the old ones, as they had the same FD and PD for fewer species. These findings do not support our initial hypothesis that increase in SD in the old patches would substantially increase the FD and PD. Contrary, the increase in SD in the old patches mainly reflects redundancy in species traits and lineages. This result reinforces growing awareness of the intricate relationship between SD and patterns of FD and PD, as previous studies have shown contrasting community assembly patterns [47, 68, 69].

By analyzing the determinants of diversity in the old and the new patches, we found that the relative importance of assembly processes changed; landscape configuration (including present-day and historical variables) dominates in the new patches, while the importance of abiotic and historical processes plays an important role in the old communities. New patches are likely to exhibit a colonization credit, as they have less species compared to the old patches, but they might gain species over time [70]. New communities encompass different functions and lineages that display the entire range of variation throughout life-history and evolutionary traits, suggesting that colonization of the new patches occurs randomly and is independent of species traits and phylogenetic affinity.

The approach of combining three sets of predictors–abiotic (environmental filtering), present-day and historical data (landscape configuration patterns)–and different diversity metrics–SD, FD and PD–clearly helped to gain a deeper understanding of the processes governing community assembly. Our study revealed that a change in SD does not always imply a change in FD and PD. However, the absence of change in FD and PD does not mean that functional and evolutionary traits stay the same, as we demonstrated with different community weights between the old and the new patches. The species co-occurring within the old and the new patches had the same functional/phylogenetic variation but different mean values.

Comparing different metrics is far beyond the scope of this paper, but we consider that the lack of functional and/or phylogenetic signal could be an artefact relating to the choice of the statistical method employed. Mean pairwise dissimilarity was selected as a measure for calculating FD and PD to reduce the complexity of our results. MPD index (i.e. mean pairwise dissimilarity) is independent from SD and more complex calculations using standardized effect sizes are not necessary [54]. However, there is no consensus on which test statistic should be used, and the methods applied profoundly affect the ecological conclusions [71]. Additionally, species are plastic and a large proportion of the variation among populations is driven by local conditions that may also impact their phylogenetic relatedness. For that reason, we assume that intraspecific variation–variation from individual to individual within a species–may better address the mechanisms driving community assembly [72, 73].

### Impacts of abiotic variables on diversity metrics

Abiotic variables affected SD, indicating that environmental filtering restricts species establishment in the new and the old patches. The lower SD in the new compared to the old ones indicates that species establishment in the new patches is limited. However, FD and PD were not related to abiotic variables in the new patches. Two possible reasons could explain the lack of abiotic signal in them. First, if species randomly colonize the new patches and species colonization is more important than establishment, abiotic variables do not differentially affect species of different traits (i.e. stochastic assembly [74]). Second, if the colonization of the new patches depends on the dispersal ability of the species, abiotic variables do not explain the establishment of the species (i.e. dispersal-driven assembly [75]). The absence of abiotic signal in the PD in any of the patches may also indicate that absence of species in sites with different abiotic conditions occurs randomly or uniformly throughout the phylogenetic tree. In other words, closely related species within the overall phylogeny do not share adaptations to the abiotic conditions. This suggests that abiotic variables are not a good surrogate for PD of dry grassland communities–environmental filtering may not determine the phylogenetic composition of dry grasslands [68, 76]–and contradicts the results of other grassland studies [43, 45, 73].

The lower SD in the new patches might be due to out-competition of the typical dry grassland species. The results for community mean analyses showed that species in the new patches tended to favor more competitive species compared to the old patches. It is possible that conditions in the new patches (e.g. flat to gentle slopes and more humid conditions) prevent the entry of typical stress-tolerant dry grasslands (i.e. species with high light and low moisture requirements) but favor more competitive species (i.e. plants with longer flowering periods, higher SLA and higher height). But caution should be taken in interpreting the CM results as the mean trait value of the species is not weighted by their abundances. Although previous grassland studies have shown that competitive exclusion is likely to reduce SD [11, 13, 47], our results did not support this hypothesis since FD was not significantly affected by the abiotic condition in the new patches, which suggests a random colonization pattern independent of specific functional traits. The low SD may thus mean that species did not have enough time to colonize the new patches [70]. This finding contradicts to previous results that found convergence patterns (i.e. communities selected by trait association) in developing grasslands [49, 77]. Contrary, environmental filtering became increasingly important in the old patches. The fact that the old patches comprised a wide range of abiotic conditions, including extremes in drought and slope, provided opportunities for dissimilar species assemblages in different conditions. For instance, slope played a much greater role than other abiotic variables in shaping dry grassland communities in the old patches, probably due to edaphic factors and drought events [78]. Generally, steeper slopes would reduce either colonization or performance of more competitive and generalist species than flatter areas [17], allowing the entry of species with more specific habitat requirements and thus translated into higher FD and PD.

### Impacts of present-day landscape configuration variables on diversity metrics

Differences in SD resulted from changes in present-day landscape configuration. This pattern is likely to have resulted from two important processes. First, SD is expected to increase with patch size as large patches contain a greater diversity of microhabitats that may reduce interspecific competition [79, 80]. Accordingly, we found that large patches hosted higher SD than small patches in the old and the new patches. This result supports findings from previous studies that have also shown higher grassland diversity as patch size increased [18–20]. Second, the (re-) colonization of patches depend on the dispersal ability of the species [16], leading to reduced SD in more isolated patches [81]. In our study, we did not find any differences in present-day isolation between the old and the new patches. Surprisingly only in the new patches, SD was negatively affected by patch isolation. A possible explanation is that species did not have enough time to establish in the isolated new patches, indicating the existence of a colonization credit [7, 75]. Under this scenario, the influence of dispersal might be declining in the old patches where communities already reached an equilibrium [18–20] and/or are dominated by long-lived species [75].

Despite the strong effect of present-day landscape configuration variables on SD, they did not affect FD in any of the patches (i.e. FD was not affected by present-day variables, neither in new nor in old patches), Small patches hosted lower SD (in both new and old patches), but they were able to maintain a constant FD as the large patches. Thus, competitive exclusion of functionally redundant species might be playing a key role in small dry grasslands regardless the age of the patch. A similar pattern was found for patch isolation. The fact that increased isolation did not reduce the trait space of the colonizing species suggests that colonization is random. This reflects, the absence of a dispersal-abiotic tradeoff selecting functional traits, in which the dispersal ability of a species is not mediated by the abiotic conditions. Therefore, contrary to hypotheses explaining macroscale patterns of diversity, abiotic conditions do not select for increased dispersal ability, which allows species to track optimal patches [82]. This could also explain the lack of phylogenetic signal in the old patches. For instance, patch area and isolation in the old dry grasslands resulted in no differences in PD, indicating that the local extirpation of species occurred uniformly throughout the phylogenetic tree. However, present-day variables had a strong effect on PD in the new patches. The large new patches showed lower PD compared to the small new patches, suggesting that the dominants of these communities recruited from a limited range of phylogenetic groups that excluded species of other groups and thus decreased PD. Similar pattern has been observed in [83] demonstrating that increased seed rain leads to reduced SD and changes in species composition leading to the dominance of species mainly from the Fabaceae family. Considering the negative effect of patch size on SD, the increase of PD in patches with small size was unexpected. Thus, the high PD found in small new patches likely reflect the colonization of distantly related species. This indicates that even small patches are important for PD and could represent important sources of evolutionary distinct species [84].

### Impacts of historical landscape configuration variables on the diversity metrics

The influence of historical effects on the three diversity metrics indicates that past processes limit the species, traits and lineages able to colonize the new and the old patches. Species respond slowly to the landscape changes [22, 23], so many grasslands reflect historical rather than present-day landscape configuration [6, 11, 13, 75]. Habitats with longer continuity have higher probabilities of being colonized due to the longer time available for species colonization [26], explaining the positive correlation of SD with patch age in the old dry grasslands. Also, we demonstrated that the overall historical effects on SD were masked by the abiotic and present-day variables in the old and the new patches, which may be related to the importance of seed bank longevity and the dispersal capacity of the species. Species are thus most likely to exhibit a colonization credit [70], generated by a mismatch between the environmental filtering that prevent their establishment or survival in the dry grassland patches and their limited dispersal abilities.

Historically connected old patches hosted an increased SD because they have been available for colonization over longer time [26], and/or because the populations are less prone to extinction [70]. Besides, we found high FD and PD in historically large connected old patches, reflecting the entry of functionally or phylogenetically dissimilar species [47]. This can happen because typical dry grassland species in the old patches may be increasingly replaced by generalist species as the propagule pressure from the new patches increases [11]. Similarly, previous results have shown a time lag in the response of grassland species to anthropogenic land use changes suggesting that slow growing and short dispersing species likely colonized the old patches long time ago [6, 75, 26]. In contrast, SD in the new patches increased in historically isolated patches (in 1843) while FD was higher in recent historically connected patches (in 1980). A possible explanation is linked to the importance of seed bank longevity promoting the co-existence of dissimilar species if the new patches are providing appropriate abiotic conditions for colonization [6, 85]. Altogether, these findings agree with previous studies showing that historical landscape configuration reflects past dispersal limitation and the absence of species cannot be compensated by dispersal from the present-day landscape [11, 75].

## Conclusions

Our study revealed that a decreasing SD in the new patches does not imply a parallel decrease in FD and PD. Despite the lower SD, FD and PD of the dry grasslands are likely to be maintained in the new patches. Thus, the function and possibly also the diversity of interactions with other organisms might be maintained. The low SD in the new patches was the result of simultaneous changes in abiotic conditions and landscape configuration (present-day variables). Interestingly, historical variables were also affecting SD, but their influence was masked by abiotic and present-day variables in the new and the old patches, respectively. Our study highlights that simultaneously evaluating the role of multiple spatio-temporal variables on different diversity facets provide better insights into the importance of environmental filtering and landscape configuration on evolutionary and life-history traits.

## Supporting information

S1 Fig Distribution of old and new dry grasslands (2009) and the spatio-temporal dynamic of potential grassland habitats within the study area in the different time periods (1843, 1954 and 1980). (DOCX)

S2 Fig Pearson correlation tests among abiotic variables. (DOCX)

S3 Fig Potential grassland habitat number and extent for different time-periods based on historical map data in the study area. (DOCX)

S4 Fig Pearson correlation tests among landscape variables (present-day and historical). (DOCX)

S5 Fig Pearson correlation tests among traits values and ecological preferences. (DOCX)

S6 Fig Phylogenetic tree for the whole species pool (n = 99) compile from DAPHNE dataset. (DOCX)

S7 Fig Venn diagrams showing the results of the variation partitioning procedure for SD, FD and PD in the ‘old’ patches and the ‘new’ patches. (DOCX)

S1 Table Values for landscape-scale variables examined in this study for different time-periods for dry grasslands and potential grassland habitat. (PDF)

S2 Table List of species used in this study. (PDF)

S3 Table Values of abiotic variables (± standard error) for old dry grasslands. (PDF)

S4 Table Database of characteristics for dry grassland patches including environmental, present-day and historical variables as well as taxonomic, functional and phylogenetic diversity indices. (TXT)
