# Supplementary material for: Abiotic, present-day and historical effects on species, functional and phylogenetic diversity in dry grasslands of different age
Source: PLoS One. 2019 Oct 15;14(10):e0223826. doi: 10.1371/journal.pone.0223826 (PMC6793948; doi:10.1371/journal.pone.0223826)

**S1 Fig. Distribution of 216 old dry grasslands (grey) and 56 new patches (red) in our study system.** The area is situated in northern Bohemia, Czech Republic (NW corner: 50°33’19” N, 14°15’6” E, SE corner: 50°29’45” N, 14°22’31” E. The figure represents a framework showing the spatio-temporal dynamic of potential grassland habitats within the study area in different time periods (1843, 1954 and 1980). For more details, see Knappová et al. [7]. Green patches are pastures, black patches are meadows and blue patches are abandoned fields.


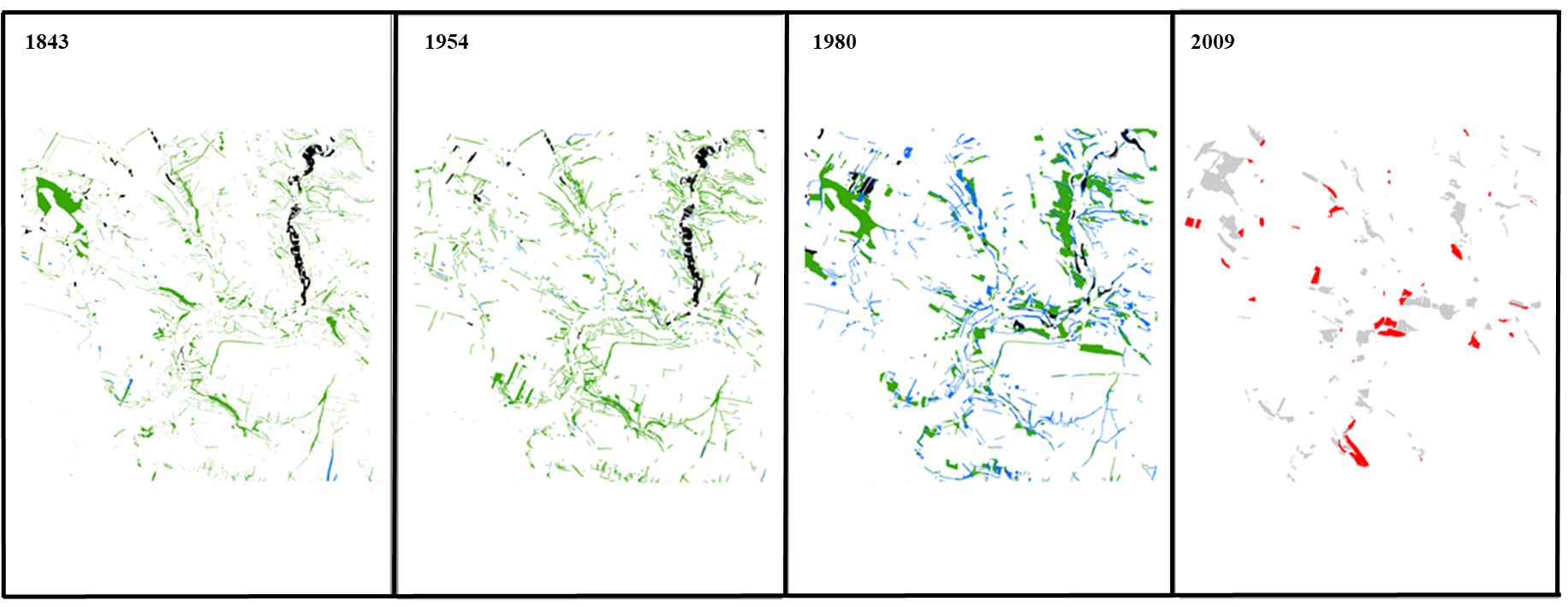

Supplement: S1 Fig — (DOCX) [file pone.0223826.s001.docx]
