# Supplementary material for: Abiotic, present-day and historical effects on species, functional and phylogenetic diversity in dry grasslands of different age
Source: PLoS One. 2019 Oct 15;14(10):e0223826. doi: 10.1371/journal.pone.0223826 (PMC6793948; doi:10.1371/journal.pone.0223826)

**S2 Fig**. **Potential grassland habitat changes in number and extent (area) in three different historical time periods (1843, 1954 and 1980) in the study area.** Color code: pastures (grey), meadows (dotted) and abandoned (black).


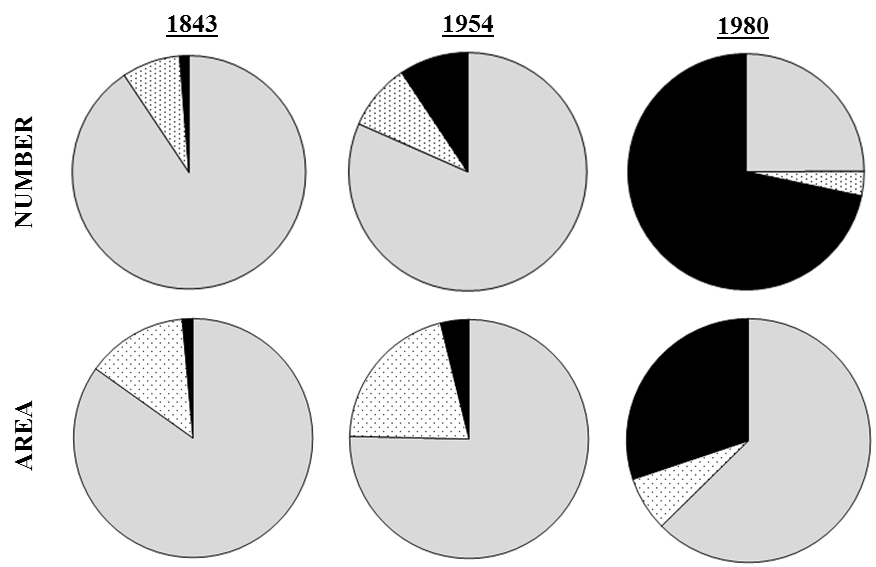

Supplement: S2 Fig — (DOCX) [file pone.0223826.s002.docx]
