# Supplementary material for: Abiotic, present-day and historical effects on species, functional and phylogenetic diversity in dry grasslands of different age
Source: PLoS One. 2019 Oct 15;14(10):e0223826. doi: 10.1371/journal.pone.0223826 (PMC6793948; doi:10.1371/journal.pone.0223826)
Supplement: S3 Fig — (DOCX) [file pone.0223826.s003.docx]

**S3 Fig. Correlation among species abiotic variables.** Coefficients of Pearson correlations and p values are presented:


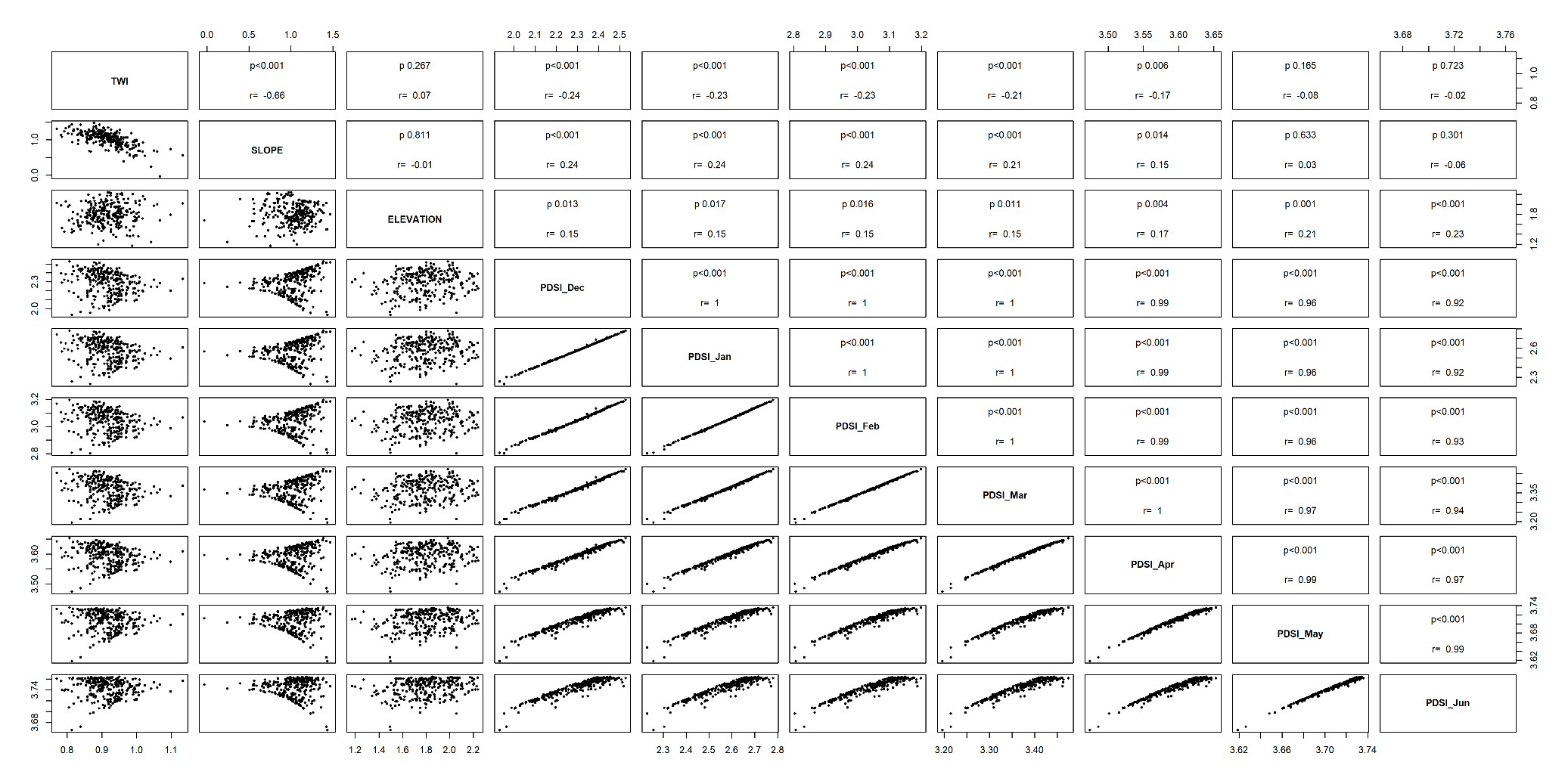


TWI, topographic wetness index; PDSI_Dec, potential direct solar irradiation in December; PDSI_Jan, potential direct solar irradiation in January; PDSI_Feb, potential direct solar irradiation in February; PDSI_Mar, potential direct solar irradiation in March; PDSI_Ap, potential direct solar irradiation in April; PDSI_May, potential direct solar irradiation in May; PDSI_June, potential direct solar irradiation in June.
