# Supplementary material for: Abiotic, present-day and historical effects on species, functional and phylogenetic diversity in dry grasslands of different age
Source: PLoS One. 2019 Oct 15;14(10):e0223826. doi: 10.1371/journal.pone.0223826 (PMC6793948; doi:10.1371/journal.pone.0223826)
Supplement: S4 Fig — (DOCX) [file pone.0223826.s004.docx]

**S4 Fig. Correlation among species traits assembled for 99 species within study and ecological preference.** Coefficients of Pearson correlations and p values are presented:


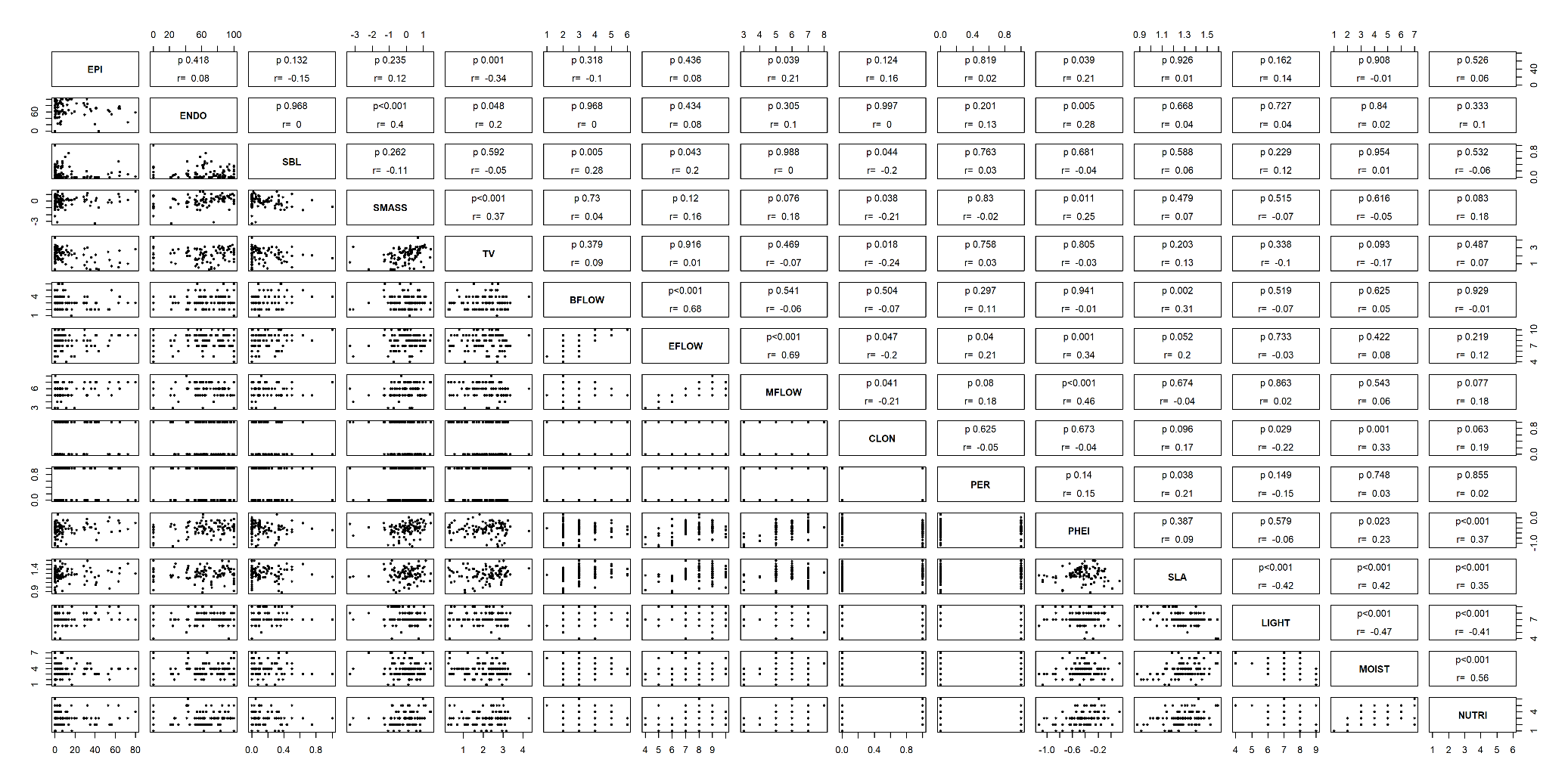


EPI, rate of epizoochory; ENDO, rate of endozoochory; SBL, seed bank longevity; SMASS, seed mass; TV, terminal velocity; BFLOW, beginning of flowering; EFLOW, end of flowering; MFLOW, duration of flowering, CLON, clonality; PER, perennial; PHEI, plant height; SLA, specific leaf area; LIGHT, Ellenberg’s light indicator value; MOIST, Ellenberg’s moist indicator value; NUT, Ellenberg’s nutrient indicator value.
