# Supplementary material for: Abiotic, present-day and historical effects on species, functional and phylogenetic diversity in dry grasslands of different age
Source: PLoS One. 2019 Oct 15;14(10):e0223826. doi: 10.1371/journal.pone.0223826 (PMC6793948; doi:10.1371/journal.pone.0223826)

**S5 Fig**. **Phylogenetic tree for the species pool (n=99) compile from DAPHNE dataset (Durka & Michalski, 2012).** Red-colored species are missing in new patches.


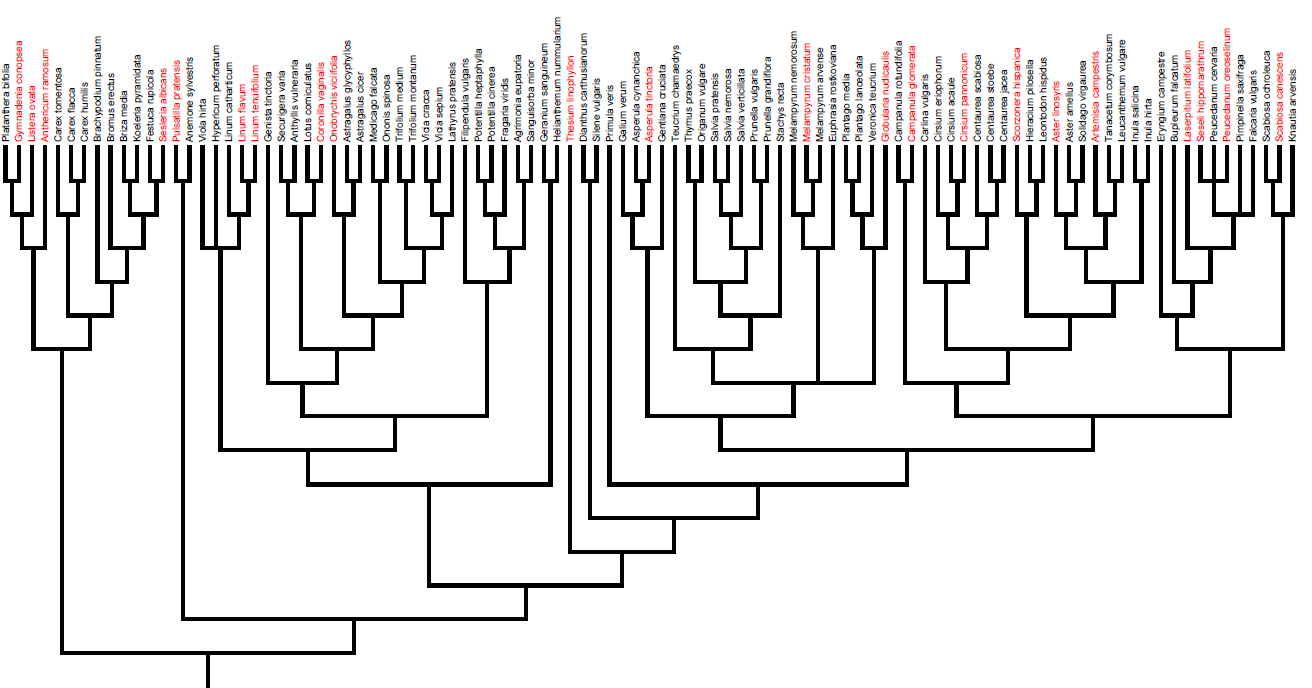

Supplement: S5 Fig — (DOCX) [file pone.0223826.s005.docx]
