# Supplementary material for: Abiotic, present-day and historical effects on species, functional and phylogenetic diversity in dry grasslands of different age
Source: PLoS One. 2019 Oct 15;14(10):e0223826. doi: 10.1371/journal.pone.0223826 (PMC6793948; doi:10.1371/journal.pone.0223826)
Supplement: S6 Fig — (DOCX) [file pone.0223826.s006.docx]

**S6 Fig. Correlation among present-day and historical landscape variables.** Coefficients of Pearson correlations are presented.


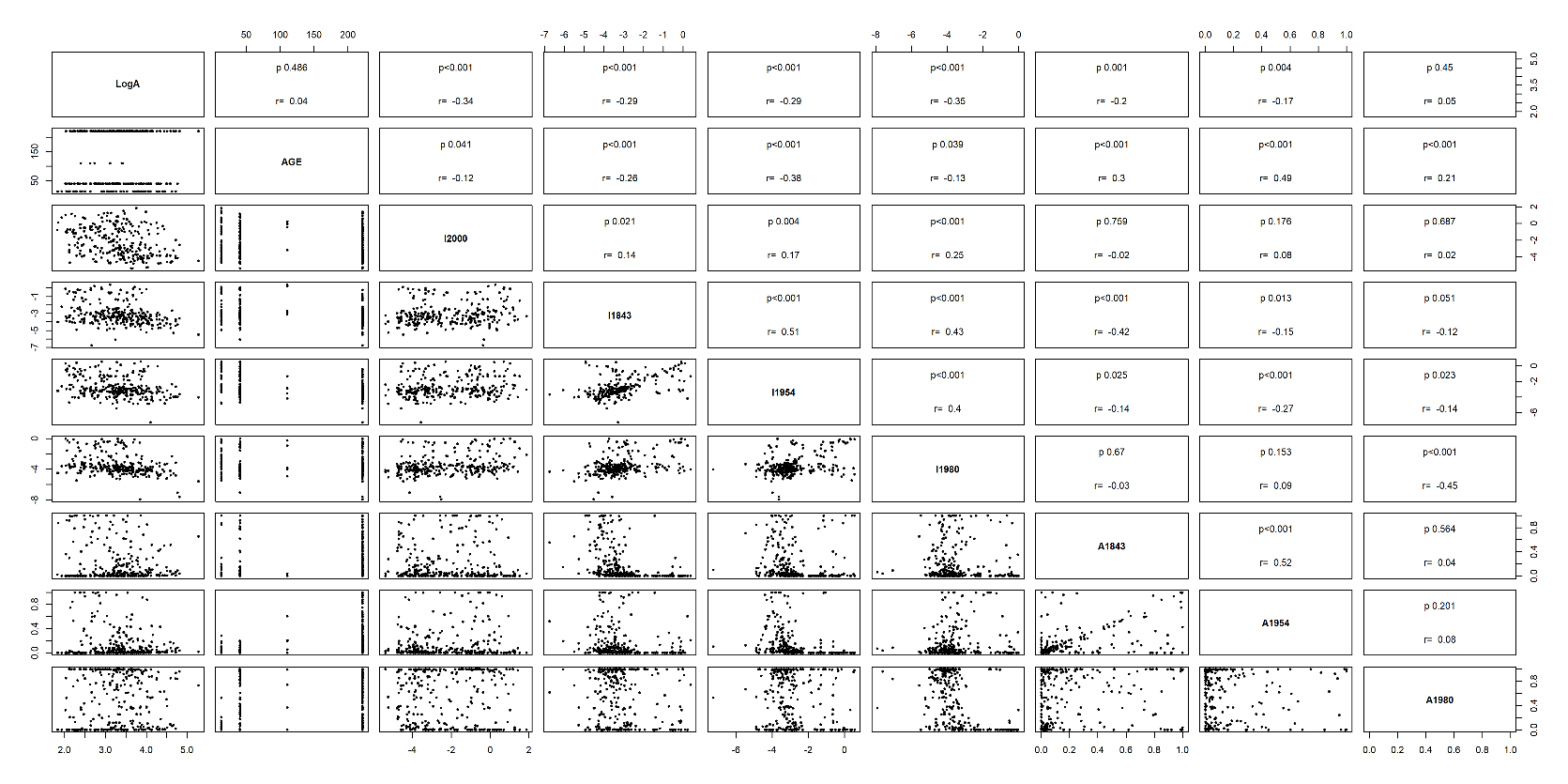


LogA, logarithm of patch area; AGE, number of years of continuous existence; I_A,_ Isolation based on the present-day area of the surrounding dry grasslands; ; I_S,_ Isolation based on the present-day species richness of the surrounding dry grasslands; I_1843_, Isolation based on the area of the surrounding potential grassland habitats in 1843; I_1954_, Isolation based on the area of the surrounding potential grassland habitats in 1954; I_1980_, Isolation based on the area of the surrounding potential grassland habitats in 1980.
