# Supplementary material for: Abiotic, present-day and historical effects on species, functional and phylogenetic diversity in dry grasslands of different age
Source: PLoS One. 2019 Oct 15;14(10):e0223826. doi: 10.1371/journal.pone.0223826 (PMC6793948; doi:10.1371/journal.pone.0223826)

**S7 Fig.** **Venn diagrams showing the results of the variation partitioning procedure for SD, FD and PD in the ‘old’ patches and the ‘new’ patches.** The total variation explained was partitioned among abiotic, present-day and historical variables with each group represented by the first two PCA axes of these variables. Values are adjusted R2 in %. Adjusted fractions of total variation explained (TVE, in %) were estimated following the procedure of [66]. Significant PCA axes are shown in boxes in each individual model.


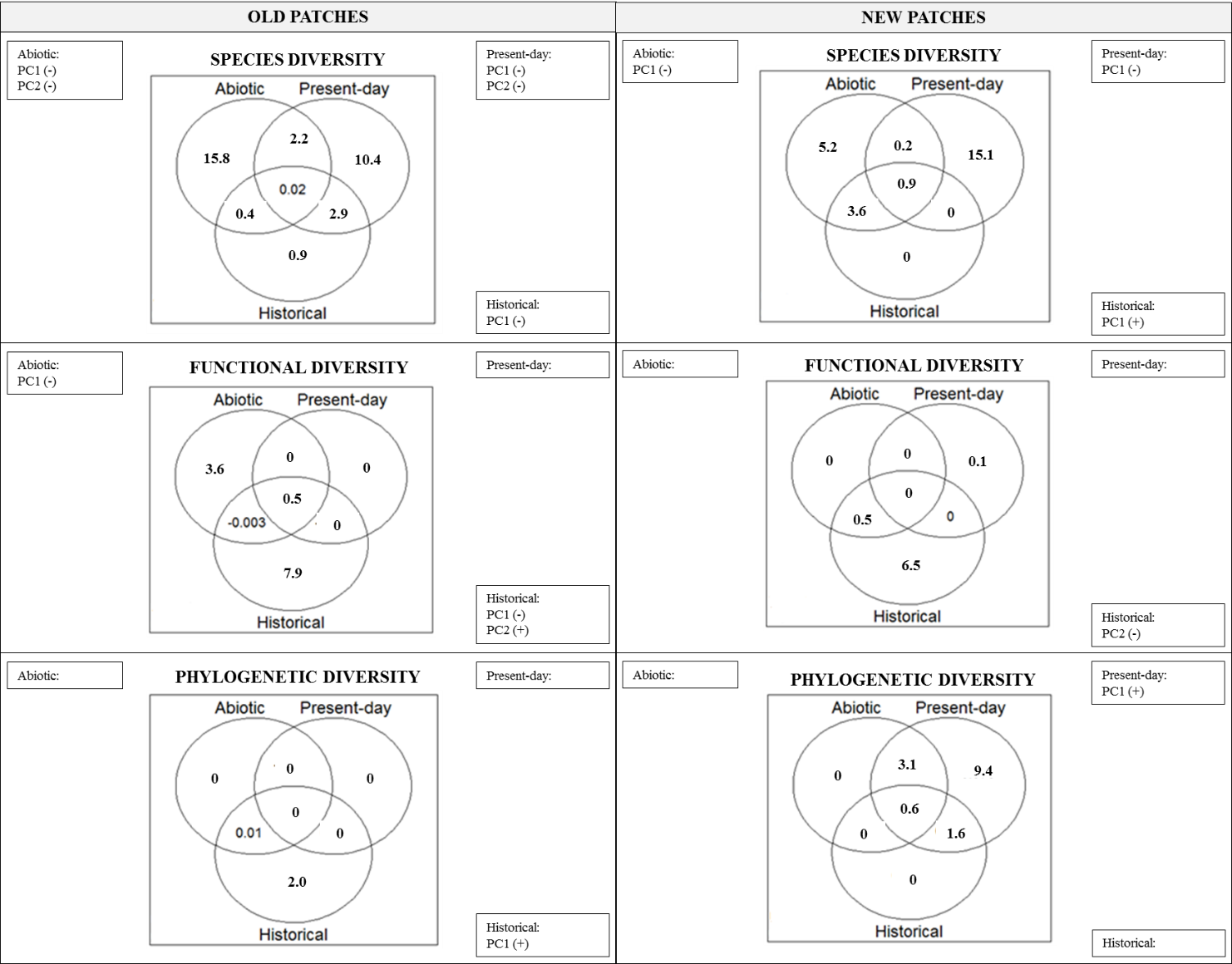

Supplement: S7 Fig — (DOCX) [file pone.0223826.s007.docx]
