# Supplementary material for: Abiotic, present-day and historical effects on species, functional and phylogenetic diversity in dry grasslands of different age
Source: PLoS One. 2019 Oct 15;14(10):e0223826. doi: 10.1371/journal.pone.0223826 (PMC6793948; doi:10.1371/journal.pone.0223826)
Supplement: S1 Table — (PDF) [file pone.0223826.s008.pdf]

S1 Table. Values for landscape-scale variables examined in this study for the four time-periods for dry grasslands and potential grassland habitat (PGH).

	1843	1954	1980	2009
Dry grasslands				
No. patches	131	137	216	216 (56)*
Mean isolation**	-3.12	-2.79	-3.55	-2.18
Potential grassland habitat				
No. PGH	4209	3404	3053	--
No. pastures	3815	2777	762	--
No. meadows	340	308	102	--
No. abandoned	54	319	2189	--
Area pastures (Ha)	255.82	165.90	315.34	--
Area meadows (Ha)	41.22	45.74	53.61	--
Area abandoned (Ha)	4.44	8.50	156.46	--
PGH area total (Ha)	301.5	220.14	585.8	--

Habitat isolation is calculated according to Eq. 1 and expressed as a mean per focal patch (values were higher for more isolated sites, when the source dry grasslands were smaller). Total habitat amount is expressed as the sum of the area of patches within 500 m buffer. *Total patches calculated as historical dry grasslands + (new patches). ** Dry grassland isolation to PGH in historical times (1843, 1954, 1980) and dry grassland isolation in present-day landscapes.
