# Supplementary material for: Abiotic, present-day and historical effects on species, functional and phylogenetic diversity in dry grasslands of different age
Source: PLoS One. 2019 Oct 15;14(10):e0223826. doi: 10.1371/journal.pone.0223826 (PMC6793948; doi:10.1371/journal.pone.0223826)
Supplement: S2 Table — (PDF) [file pone.0223826.s009.pdf]

S2 Table. List of species used in this study. [1]Species with mean trait value for specific leaf area. [2]Species with mean trait value for seed bank longevity. The taxonomy and nomenclature of species in this paper follow Tutin, T. G et al. (Eds.) 1964-1983. Flora Europaea. Cambridge, UK: Cambridge University Press.

species	Old patches	New patches
Agrimonia eupatoria	203	51
Anemone sylvestris	40	2
Anthericum ramosum	18	NA
Anthyllis vulneraria	64	3
Artemisia campestris	5	NA
Asperula cynanchica	44	1
Asperula tinctoria	23	NA
Aster amellus	24	2
Aster linosyris	15	NA
Astragalus cicer [2]	86	23
Astragalus glycyphyllos	142	33
Brachypodium pinnatum	187	42
Briza media	110	17
Bromus erectus	105	14
Bupleurum falcatum	158	29
Campanula glomerata	1	NA
Campanula rotundifolia	6	2
Carex flacca	100	13
Carex humilis	45	1
Carex tomentosa	63	9
Carlina vulgaris	65	13
Centaurea jacea	166	36
Centaurea scabiosa	156	31
Centaurea stoebe	6	1
Cirsium acaule	74	6
Cirsium eriophorum	20	10
Cirsium pannonicum [2]	9	NA
Coronilla vaginalis [2]	3	NA
Dianthus carthusianorum	36	7
Eryngium campestre	115	15
Euphrasia rostkoviana [1]	11	3
Falcaria vulgaris	107	28
Festuca rupicola	177	41
Filipendula vulgaris	8	1
Fragaria viridis	188	45
Galium verum	163	43
Genista tinctoria	26	2
Gentiana cruciata [1]	47	13
Geranium sanguineum	23	2
Globularia punctata [2]	15	NA
Gymnadenia conopsea	3	NA
Helianthemum grandiflorum [1, 2]	52	6
Hieracium pilosella	65	11
Hypericum perforatum	154	46
Inula hirta	10	1
Inula salicina	159	40
Knautia arvensis	190	49
Koeleria pyramidata	53	5

Laserpitium latifolium	2	NA
Lathyrus pratensis	51	20
Leontodon hispidus	119	28
Leucanthemum vulgare	68	11
Linum catharticum	159	36
Linum flavum [2]	10	NA
Linum tenuifolium	15	NA
Listera ovata	10	NA
Lotus corniculatus	185	38
Medicago falcata [1]	93	10
Melampyrum arvense	22	3
Melampyrum cristatum	1	NA
Melampyrum nemorosum	13	2
Onobrichis viciifolia	10	NA
Ononis spinosa	60	1
Origanum vulgare	112	33
Peucedanum cervaria	41	2
Peucedanum oreoselinum	11	NA
Pimpinella saxifraga	115	22
Plantago lanceolata	148	34
Plantago media	160	27
Platanthera bifolia	16	2
Potentilla arenaria	33	2
Potentilla heptaphylla	95	19
Primula veris	75	14
Prunella grandiflora	79	6
Prunella vulgaris	77	17
Pulsatilla pratensis [2]	3	NA
Salvia nemorosa [2]	39	3
Salvia pratensis	161	24
Salvia verticillata [2]	122	24
Sanguisorba minor	165	30
Scabiosa canescens	1	NA
Scabiosa ochroleuca	129	19
Scorzonera hispanica [2]	4	NA
Securigera varia	197	49
Seseli hippomarathrum [1, 2]	7	NA
Sesleria albicans	20	NA
Silene vulgaris	88	26
Solidago virgaurea	24	2
Stachys recta	88	14
Tanacetum corymbosum	37	6
Teucrium chamaedris	56	5
Thesium linophyllon	4	NA
Thymus praecox	74	4
Trifolium medium	150	33
Trifolium montanum	40	5
Veronica austriaca [1]	27	6
Vicia cracca	125	29
Vicia sepium	58	14
Viola hirta	53	7
