# Supplementary material for: Abiotic, present-day and historical effects on species, functional and phylogenetic diversity in dry grasslands of different age
Source: PLoS One. 2019 Oct 15;14(10):e0223826. doi: 10.1371/journal.pone.0223826 (PMC6793948; doi:10.1371/journal.pone.0223826)
Supplement: S3 Table — (PDF) [file pone.0223826.s010.pdf]

S3 Table. Minimum, maximum and mean values (± standard error) for abiotic variables for the old dry grasslands.

	MIN	MAX	MEAN	SE	threshold
TWI	5.37	16.32	8.17	0.032	16.34761
Slope	0.03	32.04	12.38	0.021	0.01167
Elevation	14.41	217.40	70.85	0.287	14.12752
PDSI_Dec	78.02	364.63	205.06	0.241	364.876
PDSI_Jan	146.83	647.12	385.55	0.404	647.532
PDSI_Feb	529.35	1658.27	1122.64	0.874	1659.142
PDSI_Mar	1469.30	3068.80	2358.69	1.263	3070.069
PDSI_Ap	2959.51	4534.27	3936.82	1.290	4535.568
PDSI_May	4128.40	5490.64	5094.62	1.043	5491.687
PDSI_Jun	4607.36	5881.48	5537.23	0.900	5882.387

TWI, topographic wetness index; PDSI_Dec, potential direct solar irradiation in December; PDSI_Jan, potential direct solar irradiation in January; PDSI_Feb, potential direct solar irradiation in February; PDSI_Mar, potential direct solar irradiation in March; PDSI_Ap, potential direct solar irradiation in April; PDSI_May, potential direct solar irradiation in May; PDSI_June, potential direct solar irradiation in June.
